# Population Kinetics and Protein Profiles of Co-Cultured Adult and Fetus Rabbit Bladder Smooth Muscle Cells

**DOI:** 10.5152/tud.2025.24120

**Published:** 2025-01-03

**Authors:** Hayrunisa Kahraman Esen, Burcu Biltekin, Mevlit Korkmaz, B. Haluk Güvenç

**Affiliations:** 1 Department of Pediatric Surgery, University of Health Sciences Faculty of Medicine, Fatih Sultan Mehmet Training and Research Hospital, İstanbul, Türkiye; 2 Department of Histology and Embryology, Atlas University Faculty of Medicine, İstanbul, Türkiye; 3 Department of Child Development, Biruni University Faculty of Health Sciences, İstanbul, Türkiye; 4 Department of Pediatric Surgery, Zonguldak Bulent Ecevit University Faculty of Medicine, Zonguldak, Türkiye

**Keywords:** Bladder smooth muscle cells, bladder tissue model, population kinetics, protein profiles

## Abstract

**Objective:**

Bladder tissue models have been developed using smooth muscle cells (SMCs) on various scaffolds to mimic bladder morphology and physiology. This study investigates the effects of co-culturing fetal and adult SMCs on growth properties and protein profiles to understand cellular interactions and population kinetics.

**Methods:**

Bladder tissue samples from 10 adult and 10 fetal New Zealand rabbits were divided into 5 groups: adult SMCs (A), fetal SMCs (F), 50%A + 50%F (A+F), 75%A + 25%F (3A+F), and 25%A + 75%F (A+3F). Population doubling time (PDT) of 3 × 10^6^ cells from each group was measured after 48 and 72 hours. Protein concentrations were estimated by spectrophotometric analysis and analyzed via SDS-PAGE gel electrophoresis. Cells exhibited typical SMC morphology, confirmed by positive staining for α-SMA and MYH11.

**Results:**

Median cell counts of single cultures were significantly higher than co-cultures (*P *< .05), but cell viability was comparable (*P *> .05). Population doubling time at 72 hours for A, F, A+F, 3A+F, and A+3F were 89.4, 92.0, 89.4, 127.9, and 145.0 hours, respectively. Protein concentrations were similar between fetal and adult co-cultures (*P *> .05). Electrophoresis at 48 hours revealed a unique 80kDa band in adult cells and a 32kDa band in co-cultured cells.

**Conclusion:**

Co-culturing resulted in increased PDT, altered protein concentrations, and changes in protein profiles, while each cell population maintained its phenotype. Fetal bladder SMCs maintained their morphology and viability when co-cultured with adult SMCs, resulting in a significant limitation in the cumulative proliferation rate. This may be dependent on alterations of protein profiles of adult and fetal SMCs promoted by rearrangements in co-cultures.

**Main Points**

- We have previously demonstrated that the PDT and S-phase fraction of adult bladder SMCs were markedly shorter than those of fetal-derived cells. Our study investigates how various potential mixing proportions in a co-culture condition could influence the outcome.
- The interaction in the co-culture condition confirms that utilizing fetal bladder SMCs is likely to induce a decline in overall PDTs. Co-culturing seems to induce a state of cell cycle arrest or prolongation of specific cell cycle phases, down-regulating overall cell proliferation.
- The characterization and identification of the proteins modulating the cell cycle might be worthy of further investigation using high-throughput proteomic approaches.

## Introduction

Bladder augmentation is a challenging surgical procedure and is exclusively offered in pediatric urology. Therefore, experimental and clinical studies have focused on tissue engineering of the bladder.^1^ These studies have mainly covered the in vitro expansion of bladder tissues and the evaluation of their use in the bladder reconstruction.^2-4^ Tissue engineering of the bladder wall involves obtaining a biopsy, expanding cells, seeding them over a synthetic or natural matrix, and implanting the cell-matrix composite back into the host.^5^ Experimental use of adult and fetal tissue replacements for a deficient or malfunctioning bladder has been reported separately;^6-9^ however, the interaction of these adult and fetal cell populations has not been studied sufficiently. We have previously demonstrated that fetal bladder smooth muscle cells (SMCs) emerged from the explants early after seeding onto the dishes. However, the population doubling time (PDT) and S-phase fraction of adult bladder SMCs were markedly shorter than those of fetal-derived cells, suggesting a preliminary investigation of these 2 cell populations.^7^ Here, we co-cultured adult and fetal rabbit bladder SMCs and investigated the effects of different gradients of co-cultures on cell phenotype, proliferation, population kinetics, and protein profiles. We hypothesized that co-culture of fetal and adult bladder SMCs may affect the doubling time due to the effect of fetal cells on adult cells.

## Material and Methods

### Animals

Adult and fetal (at 27-30 days gestational age) New Zealand white rabbits were used as donors. A total of 10 healthy female rabbits weighing 3500-4000 g and 10 healthy female rabbits weighing 3000-3500 g in the last 5 days of pregnancy were retrieved from Uludağ University Faculty of Medicine DETAM animal laboratory. The protocol of the study was approved by the Ethical Commission for Research from Kocaeli University School of Medicine (1.11.2002 / AEK-292/4). All animal experiments complied with the ARRIVE guidelines and were carried out in accordance with EU Directive 2010/63/EU.

### Bladder Biopsy

Surgical procedures for bladder biopsies were performed under intramuscular xylazine (5 mg/kg) and ketamine (25 mg/kg) anesthesia. Tissue samples were obtained as a bladder muscle biopsy from adults and as a whole bladder from fetuses. Five-to-six fetuses were removed from each pregnant rabbit. The fetal bladder was reached by opening the fetal abdomen under ether anesthesia; the bladder was excised and placed in sterile 0.9% isotonic solution. Each fetus was sacrificed with ether. The specimens were processed within one hour after surgery.

The same procedure was applied to adult female rabbits, and approximately 2 cm^2^ muscle tissue was excised without perforating the mucosa and taken into a sterile isotonic solution. After the surgical procedure, adult female rabbits were administered intramuscular ampicillin-sulbactam (50 mg/kg) and novalgine (10 mg/kg) and placed in their cages.

### Cell Culture

Dulbecco’s modified eagle medium (DMEM), supplemented with 10% fetal calf serum (Sigma Chemical Co, St. Louis, MO), 100 U/mL penicillin, and 0.1 mg/mL streptomycin, was used as culture media. The bladder wall was stripped of its mucosa and placed in culture medium; detrusor smooth muscle samples were cut into small pieces with scissors or lancets under sterile conditions. Each suspension was placed onto dishes for explant culture, and 10 mL of medium was added. Cells were grown at 37°C in an incubator with a humidified atmosphere of 5% CO_2_ in air. When culture dishes reached semi-confluence, cells were harvested after treatment with 0.025% trypsin-0.2% EDTA (Serva) solution.

### Immunocytochemistry

The isolated cells were identified as SMCs at the second passage by immunostaining for α-smooth muscle actin (α-SMA) and myosin heavy chain 11 (MYH11) using monoclonal antibodies against these proteins (Sigma). In this indirect immunoperoxidase procedure (Biogenex, USA), the peroxidase activity was visualized by incubation in 3-amino-9-ethyl-carbazole (AEC). The slides were examined by light microscopy (Nikon, Tokyo, Japan). Control sections were incubated in the absence of the primary antibody.

### Co-cultures

After adult and fetal cells were harvested and resuspended, the cell numbers and viability of all samples were determined by trypan-blue exclusion assay, and the cells were counted by hemocytometer under an inverted microscope. Reaching 3 × 10^6^ cell numbers of adult and fetal bladder SMCs in 1 mL medium, the adult and fetal cells were mixed one by one and randomly selected at 50:50, 75:25, and 25:75 ratios in co-culture medium and inoculated into a material called flask consisting of wells. These ratios were selected randomly to observe a broad range of interactions between the adult and fetal SMCs in order to estimate how varying dominance would affect the population kinetics, and protein profiles of one cell type over the other. Specifically, the 25% vs 75% proportions enable us to examine both scenarios according to the state of predominance among the co-cultured cells. Groups were determined as follows: individually isolated cells from adult SMCs (A) and fetal SMCs (F), co-culture composed of equal numbers of cells from both cultures (A+F), co-culture composed of cells in 3 times the amount of either A and F cells (75% A+F 25% and A 25%+75% F).

In order to determine the PDT of the cells for the 48th and 72nd hours of incubation, growing cells were harvested by trypsinization and re-suspended. The total cell count was determined by multiplying the number of cells obtained by the volume of the solution as described previously.^7^

All experiments with cell cultures were repeated 3 times.

### Protein Extraction and Quantification

In all groups, to extract both cytoplasmic and nuclear proteins, the cell suspensions were processed using a total protein extraction kit (MPER Pierce, USA). Initially, cells were collected from the culture flasks, washed twice with ice-cold PBS, detached by a cell scraper and centrifuged at 2500 rpm at 4°C. The pellets were weighed as 200 mg and mixed with MPER solution to lyse the cells. The lysed cells were centrifuged at 14 000×g for 15 min, and the supernatant was collected.

Total protein concentration was determined using the Bradford dye binding assay with a ready-to-use kit (BioRad, USA). Different concentrations of serum albumin (BSA) were used as protein standards. Briefly, 0.1 mL of protein standard or proteins with unknown concentrations, 0.1 mL of distilled water, and 5 mL of diluted dye were mixed and incubated for 5 minutes at room temperature. Following incubation, OD_595_ values were recorded with a spectrophotometer (Shimadzu UV1208), and a standard curve was plotted for the determination of protein amounts in unknown solutions. The spectrophotometric results and total protein concentrations were compared between study groups and estimated their protein concentrations at 48th and 72nd hours (Table 1).

### SDS-Polyacrylamide Gel Electrophoresis

Sodium Dodecyl Sulfate-Polyacrylamide Gel Electrophoresis (PAGE) was performed according to the protocol reported by Laemmli et al.^10^ Twelve percent separating and 4% stacking gel concentrations were used, and the gels were run for 50 minutes at 180 volts. After the runs were completed, the gels were fixed in a solution composed of 40% methanol and 4% acetic acid for 30 minutes, then the proteins were stained with silver stain (BioRad, USA) for visualization.^11^

### Statistical Analysis

SPSS for Windows 10.0 (SPSS Inc.; Chicago, IL, USA) program was used for statistical analysis. The descriptive statistical data included the mean, standard deviation, median, and range. Normality was tested by the Kolmogorov-Smirnov test. Kruskal Wallis test was used for the comparison of quantitative variables that did not show normal distribution, and Mann–Whitney U-test was used to determine the group that caused the significance. The Wilcoxon sign test was used for in-group comparisons. In the comparison of qualitative data, the chi-square test was used. The results were evaluated at the 95% confidence interval and the significance level of *P *< .05.

## Results

**Figure 1.**
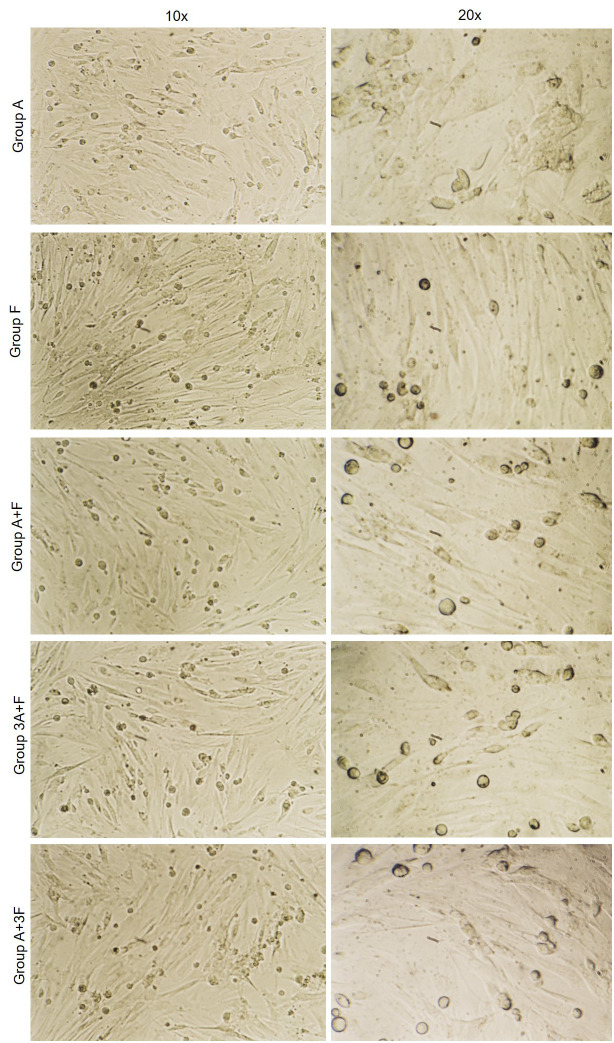
Light microscopic images of adult smooth muscle cells (SMCs) (A), fetal SMCs (F), 50% A + 50% F (A+F), 75% A + 25% F (3A+F) and 25% A + 75% F (A+3F).

The monolayer formation of fetal bladder SMCs collected from tissue cultures was observed following 3-4 days of incubation, whereas that of adult bladder SMCs was observed following 5-6 days of incubation. Cell morphology was not affected by the cultivation method used, whether as single or co-culture. Light microscopy analysis of bladder SMCs of all groups revealed the typical morphological features of SMCs characterized by the presence of spindle-shaped morphology and centrally located round-to-oval nuclei (Figure 1).

**Figure 2.**
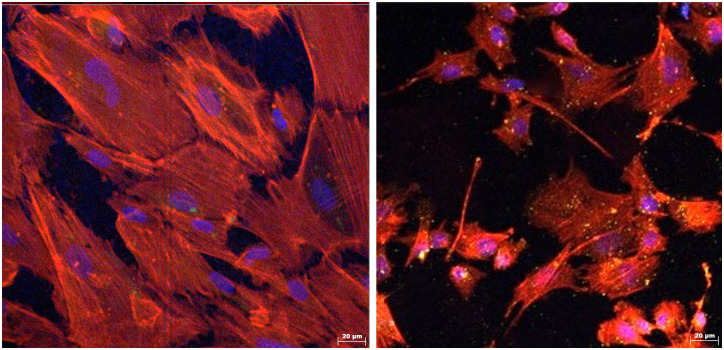
Images of immunostaining show the expression of alpha smooth muscle actin (α-SMA, colored green) and myosin heavy chain 11 (MYH11, colored yellow) in the adult bladder smooth muscle cells (left image) and the fetal bladder smooth muscle cells (right image). Nuclei were counterstained with 4α,6-diamidino-2-phenylin-dole (DAPI, colored blue), and the cytoskeleton was visualized with phalloidin (colored red).

Immunocytochemistry by staining with α-SMA and MYH11 antibodies showed the characteristics of SMCs in cell cultures (Figure 2).

The counts of bladder adult and fetal SMCs did not differ among the 5 groups including single and co-cultures incubated for 48 hours. However, the median cell counts of single cultures of fetal and adult SMCs were significantly higher than those of the 3 co-cultures (Table 2). On the other hand, the viabilities of bladder SMCs of all groups were comparable, ranging between 95.16% and 97.75% at 48 hours and 96.28% and 99.07% at 72 hours of incubation (*P *> .05) (Table 1).

**Figure 3.**
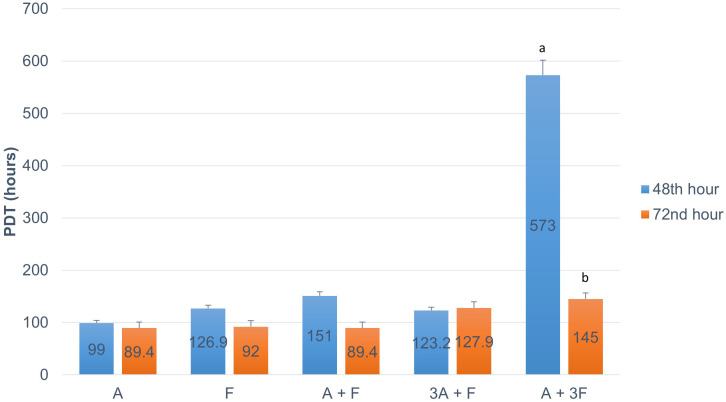
Population doubling time of cell populations of adult smooth muscle cells (A), fetal smooth muscle cells (F), 50% A + 50% F (A+F), 75% A + 25% F (3A+F) and 25% A + 75% F (A+3F). (a) *P *< .001 vs PDTs of other groups incubated for 48 hours. (b) *P* < .05 vs PDT of cells in the A+3F group incubated for 48 hours. All experiments with cell cultures were repeated 3 times. The numbers on bars represent the mean of PDTs, and error bars represent the standard error.

Population doubling time measured from the adult and fetal bladder SMCs were comparable (*P *> .05). However, PDTs measured from the co-culture of A+3F SMCs were significantly higher than those at corresponding incubation periods in other groups (*P* < .05) (Figure 3).

**Figure 4.**
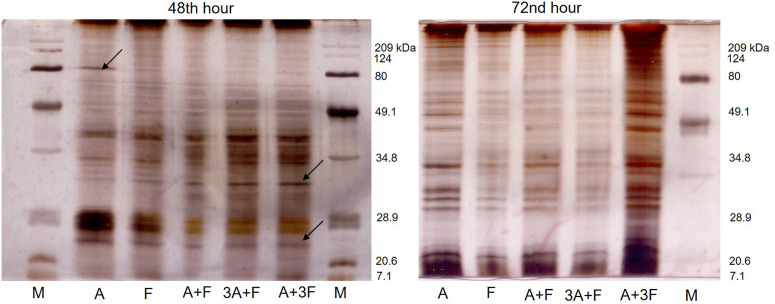
Protein gel electrophoresis of protein extracts from adult smooth muscle cells (A), fetal smooth muscle cells (F), 50% A + 50% F (A+F), 75% A + 25% F (3A+F) and 25% A + 75% F (A+3F) groups after 48 and 72 hours of incubation. Arrows are showing discrete protein bands.

Total protein analysis indicated satisfactory protein extraction with no evidence of degradation during the experiment. A protein band consistently detected at a molecular weight of 26 kDa in all protein extracts of the groups served as a control for normalizing protein loadings and ensuring equal loading for comparative analysis. Additionally, a protein band with an average molecular weight of 80 kDa was observed in cell-free extracts obtained from adult SMC cultures after 48 hours of incubation. However, an identical band was not observed in either single cultures or co-cultures after 72 hours of incubation. Instead, a heavily stained protein band in the 30 kDa range was consistently present in all cell-free extracts obtained from co-cultures after 48 hours of incubation, albeit with weak intensity in extracts from adult or fetal SMC cultures alone. Protein profiles of SMCs harvested after 72 hours of incubation were comparable among the five groups (Figure 4).

## Discussion

Utilization of autologous cells is considered a valuable strategy in treating bladder diseases via tissue engineering.^1^ This concept includes obtaining cells from various sources, in vitro expansion followed by transferring onto a fabricated matrix, and finally replacement for bladder augmentation. Successful results from experimental or clinical studies concerning bladder regeneration have been reported using multi-originated cells, adult or fetal tissues, and recently embryonic or mesenchymal stem cells, the latter being said to induce smooth muscle differentiation.^12,13^ The engineered bladder was reported to gain normal function in time.^1,14,15^ It was also claimed that the employment of autologous fetal tissue may carry the advantages of a higher proliferation rate and capacity for tissue engineering.^6^ Studies concerning co-cultures of bladder SMCs regarding their interactions with urothelial cells or stem cells are rare but increasingly found in the literature.^16-19^ In a phase II study in children and adolescents with spina bifida, the urothelial and smooth muscle cells were grown ex vivo and seeded onto a biodegradable scaffold to form a regenerative augment as the foundation for bladder tissue regeneration. However, the autologous cell-seeded biodegradable scaffold did not improve bladder compliance or capacity.^20^

On the other hand, little is known about the interactions of individual characteristics of fetal and adult cell populations. Therefore, we suggested that in vitro examination of the phenotypic and functional characteristics of co-cultured cell populations should be elucidated before using them as a graft. In the present study, we investigated the effects of co-culturing fetal and adult SMCs on the growth properties and protein profiles. Here, the proliferation rates of the single-cell cultures of fetal and adult bladder SMCs were comparable at both 48 and 72 hours of incubation. However, the interaction of adult and fetal SMCs in the co-culture condition resulted in a decrease in total cell proliferation. The findings suggest that fetal bladder SMCs may not be an optimal choice to induce a higher proliferation rate as expected, but rather decrease the PDTs in co-cultures with adult SMCs.

In our previous study, we investigated and compared the growth properties of rabbit bladder SMCs derived from the single cell cultures of fetal and adult rabbit bladder in vitro. We reported that the fetal cells emerged from tissue explant earlier than the adult cells, but their PDT and thymidine labeling index (TLI) after the second passage showed lower proliferation rates when compared to those of the adult cells.^7^ This result was explained by the extensive plasticity of the phenotype of SMCs, as it was reported previously in vascular muscle cells.^21^ Results of our other similar study concerning esophageal SMCs revealed no significant differences in the tissue mass qualities of fetal and adult cells. On the other hand, fetal esophageal SMCs were once again found to proliferate at a slower pace than adults, consistent with our previous study.^22^

Stromal cell contamination is a challenging problem for selective cell isolation. The mucosa and epithelium should be meticulously peeled away from the muscle layer to minimize the contamination of epithelial cells in culture. As reported in initial studies, cell type-specific medium and careful tissue dissection may improve the isolation and growth of specific cell population.^7,23^ Fibroblast proliferation is prevented by including D-valine in the culture medium. L-valine is an essential amino acid required for growth; an enzyme absent in fibroblasts but present in smooth muscle cells.^24^ SMCs obtained from host bladder may be maintained in culture for longer periods over several passages, following initial establishment in the culture medium. These cells express specific cell markers that respond to stimulation by acetylcholine as contraction.^25^ We selected DMEM that contained high glucose and calcium concentrations and found that cultures grew with low stromal contamination and were stained over 90% positive with SMC marker protein antibodies. In contrast to enzymatic digestion protocols, tissue explant techniques, including small pieces of tissue, provide sufficient cell cultivation in a brief time. Sharma et al^26^ reported cell heterogeneity under conventional methods utilized for bladder SMC isolation. This is essential in terms of tissue engineering under specific cell type requirements for the establishment and maintenance of tissue grafts in vivo. In the present study, all cells displayed typical SMC morphology, detected by positive staining for ɑ-SMA and MYH11.

The available quantity of fetal or adult cells may vary in regenerative medicine or tissue engineering applications.^27^ Preliminary experiments and a review of relevant literature indicate that significant interactions and observable differences in cell behavior and protein expression might occur at these specific ratios.^28,29^ Our study provided insights into how different mixing proportions could influence the outcomes. The chosen randomized ratios of co-cultures may reflect the various potential clinical scenarios in terms of unequal proportional seeding of cells from different sources. The 50%:50% ratio serves as a control to compare balanced interactions, while the 25% vs. 75% and 75% vs 25% ratios help us investigate the effects of an imbalance in cell populations. These ratios provided a manageable and reproducible approach for our experimental design, ensuring that we could consistently and accurately measure PDT, protein concentrations, and protein profiles across different groups. We believe that exploring these specific proportions may provide valuable insights into the interaction dynamics between adult and fetal bladder SMCs, contributing to our understanding of cellular behavior in co-culture systems.

We observed that the median cell counts of single cultures were significantly higher than those of co-cultures, while cell viability remained comparable across all groups. This finding indicates a key aspect of the possible interaction between co-cultured adult and fetal bladder SMCs. Population doubling time at 48 and 72 hours was shorter in single cultures compared to co-cultures. The increase in PDT observed in co-cultures compared to single cultures suggests alterations in cell cycle dynamics. Co-culturing might induce a state of cell cycle arrest or prolongation of specific cell cycle phases, leading to slower overall cell proliferation. In a co-culture environment, adult and fetal SMCs may compete for essential nutrients and growth factors. This competition can limit the resources available to each cell type, thereby reducing the overall cell proliferation rate compared to single cultures where such competition is absent. Despite the lower cell counts in co-cultures, cell viability remained high and comparable among all groups. This indicates that while proliferation rates are affected, the overall health and survival of the cells are not compromised. This could be due to adaptive mechanisms that ensure cell survival under varying growth conditions.

The reduced cell counts in co-cultures suggest that direct interactions between adult and fetal SMCs may lead to mutual growth inhibition. This phenomenon could be mediated by several mechanisms, including contact inhibition, which might trigger signaling pathways that inhibit proliferation. Another mechanism is paracrine signaling. We may postulate that co-cultured cells induce secretion of growth factors, cytokines, or other signaling molecules that negatively regulate cell proliferation. These paracrine signals can have an inhibitory effect on neighboring cells. Our findings suggest that co-culturing adult and fetal SMCs may have a negative impact on proliferation rates while preserving cell viability. This knowledge is valuable for designing optimal cell combinations and conditions for tissue engineering applications, where controlled growth is essential.

Bladder muscle cells from different origins (normal, myelomeningocele, exstrophy) were analyzed for specific proteins in the literature, where consistent expression of these proteins regardless of the origin was present within the culture media. The muscle cell phenotype was preserved with multiple passages in vitro.^30,31^ These proteins function in cell growth, cell cycle regulation, intracellular kinase networks, calcium binding, and immunological pathways. However, some reports showed that the cultivation of dispersed vascular smooth muscle cells invariably caused rapid modulation from the contractile to the proliferative phenotype. This change involved loss of contractibility, decreased contractile protein content, and increased expression of rough endoplasmic reticulum.^32,33^ Therefore, we examined the protein profiles of adult and fetal SMCs and their compositions.

Variations in the protein concentrations, which may indicate differential gene expression in fetal and adult bladder tissues, have been observed in our study as well.^34^ Protein concentrations were slightly higher in fetus and adult co-cultures. Total protein analysis showed different protein profiles among adult SMCs and co-cultured SMCs, especially at 48 hours of incubation. Co-culturing of adult and fetus rabbit bladder SMCs resulted in an increase in the PDT, changes in protein concentrations, and alterations in protein profiles while each cell population retained its phenotype. The strongly stained protein band at around the 30 kDa range was present in the cell-free extracts obtained from the co-cultures, which may be the reason for a prolonged doubling time. The 30 kDa could be galectin or aquaporin, which come from the bladder epithelium. The protein band at around 80 kDa could be L-caldesmon, PKC gamma (regulators of bladder contractility), or LPP (unknown function). Future sequencing data may lead to interesting mechanistic questions about what is happening to the adult rabbit bladder SMCs and which intracellular signaling proteins with protein-coupled receptors may play a significant role in this phenomenon.

As noted, our results indicated that distinct protein bands were not observed between the samples after 72 hours of incubation. This finding suggests that the alterations in protein profiles induced by co-culturing adult and fetal SMCs are indeed dynamic and may only be prominent during the early stages of co-culture. The initial phase of co-culture likely involves significant cellular interactions and signaling events as the adult and fetal SMCs adjust to their new environment. These interactions could lead to the transient expression of specific proteins, which might not be sustained as the cells reach a more stable state. Over time, the cells might adapt to the co-culture conditions, leading to a convergence in their protein expression profiles. This adaptation could result in the disappearance of distinct protein bands as the cells find a new equilibrium. Moreover, it is possible that the initial proteomic changes are part of a short-term response mechanism. Once the cells have undergone initial adjustments, their protein expression profiles might stabilize, reflecting a more consistent state of cellular function and phenotype. Further research will be essential to fully understand the implications and mechanisms behind these observations.

The limitations of the study are the lack of methodology for showing the minimal stromal or fibroblast activity in the cell cultures and the lack of characterization of the protein at 26 kDa used for normalization and the 80 kDa observed in cell-free extract obtained from adult SMC cultures. Another limitation is the collection of the fetal cells after 3-4 days and adult cells after 5-6 days, which were determined by monolayer formation. This may be due to a resultant reduction in fetal cell doubling time while the cells were approaching confluence. It may have also be due to continuing alterations in the observed protein and metabolite profiles. Moreover, we do not have information about the origin of the doubled cells in cultures, although we performed IHC to characterize the smooth muscle cells. Our findings would become more enlightening if the difference in the proliferation of single vs. co-cultures could be confirmed by gene expression studies such as real-time PCR or Western blotting.

The fetal bladder SMCs appear to sustain their morphological characteristics and viability even when cultivated together with adult SMCs, resulting in a significant limitation in the cumulative proliferation rate. This limitation may be dependent on the alterations of protein profiles of adult and fetal SMCs promoted by the rearrangements in the co-cultures. The final molecular and metabolic products of co-cultures of adult and fetal SMCs and the characterization of the proteins modulating the cell cycle might be worthy of further investigation using high-throughput proteomic approaches. Down-regulation and/or up-regulation of these specific proteins or other molecules may aid in slowing down the PDT and altering the population kinetics under uncontrollable cellular mechanisms. Our findings suggest new insights into the interactions of fetus and adult rabbit bladder SMCs to provide an opinion for fetal cell therapy.

## Data Availability Statement:

The data of this study is available upon request to the corresponding author.

## Figures and Tables

**Table 1.** Spectrophotometric Values and Total Protein Concentrations of Adult Smooth Muscle Cells (SMCs) (A), Fetal Smotth Muscle Cells (F), 50% A + 50% F (A+F), 75% A + 25% F (3A+F) and 25% A + 75% F (A+3F) After 48 and 72 Hours of Incubation

	OD_595nm_		Protein (mg/mL)	
	48th hour	72nd hour	48th hour	72nd hour
A	0.357	0.6	0.309	0.674
F	0.354	0.68	0.305	0.793
A + F	0.532	0.518	0.572	0.551
3A + F	0.491	0.599	0.51	0.672
A + 3F	0.496	0.525	0.518	0.561 ^1^Optical Density at 595 nm

**Table 2.** The Counts of Bladder Adult Smooth Muscle Cells (A), Fetal Smooth Muscle Cells (F), 50% A + 50% F (A+F), 75% A + 25% F (3A+F) and 25% A + 75% F (A+3F) after 48 and 72 Hours of Incubation

Groups	Cell count				*P*
	48th hour		72nd hour		
	Mean	Median	Mean	Median	
A	422500	422500	523333	532500	.102
F	391667	365000	518333	527500	.285
A+F	377500	377500	523333	505000	.109
3A+F	396667	410000	445000	430000*	.109
A+ 3F	320833	320000	425833	427500*	.109
P value	0.109		**0.041**		

All experiments with cell cultures were repeated 3 times. Descriptive data was presented as the median for statistical comparison.

*P = .046 vs A group, F group, A+F group.

## References

[1] Casarin M Morlacco A Dal Moro F . Tissue engineering and regenerative medicine in pediatric urology: urethral and urinary bladder reconstruction. Int J Mol Sci. 2022;23(12):6360. (10.3390/ijms23126360) 35742803 PMC9224288

[2] Kimuli M Eardley I Southgate J . In vitro assessment of decellularized porcine dermis as a matrix for urinary tract reconstruction. BJU Int. 2004;94(6):859 866. (10.1111/j.1464-410X.2004.05047.x) 15476523

[3] Horst M Eberli D Gobet R Salemi S . Tissue engineering in pediatric bladder reconstruction-The road to success. Front Pediatr. 2019;7:91. (10.3389/fped.2019.00091) 30984717 PMC6449422

[4] Steins A Dik P Müller WH , et al. In vitro evaluation of spider silk meshes as a potential biomaterial for bladder reconstruction. PLoS One. 2015;10(12):e0145240. (10.1371/journal.pone.0145240) 26689371 PMC4687005

[5] Gasanz C Raventós C Morote J . Current status of tissue engineering applied to bladder reconstruction in humans. Actas Urol Esp (Engl Ed). 2018;42(7):435 441. (10.1016/j.acuro.2017.11.005) 29336836

[6] Fauza DO Fishman SJ Mehegan K Atala A . Video fetoscopically assisted fetal tissue engineering: bladder augmentation. J Pediatr Surg. 1998;33(1):7 12. (10.1016/s0022-3468(98)90350-5) 9473089

[7] Korkmaz M Güvenç BH Bilir A , et al. Isolation and culture of adult and fetal rabbit bladder smooth muscle cells and their interaction with biopolymers. J Pediatr Surg. 2003;38(1):21 24. (10.1053/jpsu.2003.50003) 12592612

[8] Leonhäuser D Vogt M Tolba RH Grosse JO . Potential in two types of collagen scaffolds for urological tissue engineering applications - Are there differences in growth behaviour of juvenile and adult vesical cells? J Biomater Appl. 2016;30(7):961 973. (10.1177/0885328215610824) 26475852

[9] Lilien OM Camey M . 25-year experience with replacement of the human bladder (Camey procedure). J Urol. 2017;197(2S):S173 S179. (10.1016/j.juro.2016.10.106) 28012761

[10] Laemmli UK . Cleavage of structural proteins during the assembly of the head of bacteriophage T4. Nature. 1970;15;227(5259):680 685. (10.1038/227680a0) 5432063

[11] Bradford MM . A rapid and sensitive method for the quantitation of microgram quantities of protein utilizing the principle of protein-dye binding. Anal Biochem. 1976;72:248 254. (10.1006/abio.1976.9999) 942051

[12] Pokrywczynska M Rasmus M Jundzill A , et al. Mesenchymal stromal cells modulate the molecular pattern of healing process in tissue-engineered urinary bladder: the microarray data. Stem Cell Res Ther. 2019;10(1):1 7. (10.1186/s13287-019-1211-x) 31196214 PMC6567623

[13] Wang Y Yang B Hu P , et al. The role of gap junctions in the generation of smooth muscle cells from bone marrow mesenchymal stem cells. Dis Markers. 2022;12. (10.1155/2022/3025306) PMC9391152 35990247

[14] Atala A Bauer SB Soker S Yoo JJ Retik AB . Tissue-engineered autologous bladders for patients needing cystoplasty. Lancet. 2006;367(9518):1241 1246. (10.1016/S0140-6736(06)68438-9) 16631879

[15] Becker C Jakse G . Stem cells for regeneration of urological structures. Eur Urol. 2007;51(5):1217 1228. (10.1016/j.eururo.2007.01.029) 17254699

[16] De Coppi P Callegari A Chiavegato A , et al. Amniotic fluid and bone marrow derived mesenchymal stem cells can be converted to smooth muscle cells in the cryo-injured rat bladder and prevent compensatory hypertrophy of surviving smooth muscle cells. J Urol. 2007;177(1):369 376. (10.1016/j.juro.2006.09.103) 17162093

[17] Frimberger D Morales N Gearhart JD Gearhart JP Lakshmanan Y . Human embryoid body-derived stem cells in tissue engineering-enhanced migration in co-culture with bladder smooth muscle and urothelium. Urology. 2006;67(6):1298 1303. (10.1016/j.urology.2005.12.005) 16750247

[18] Lakshmanan Y Frimberger D Gearhart JD Gearhart JP . Human embryoid body-derived stem cells in co-culture with bladder smooth muscle and urothelium. Urology. 2005;65(4):821 826. (10.1016/j.urology.2004.11.022) 15833554

[19] Liao W Yang S Song C Li X Li Y Xiong Y . Construction of ureteral grafts by seeding bone marrow mesenchymal stem cells and smooth muscle cells into bladder acellular matrix. Transplant Proc. 2013;45(2):730 734. (10.1016/j.transproceed.2012.08.023) 23498814

[20] Joseph DB Borer JG De Filippo RE Hodges SJ McLorie GA . Autologous cell seeded biodegradable scaffold for augmentation cystoplasty: phase II study in children and adolescents with spina bifida. J Urol. 2014;191(5):1389 1395. (10.1016/j.juro.2013.10.103) 24184366

[21] Cook CL Weiser MC Schwartz PE Jones CL Majack RA . Developmentally timed expression of an embryonic growth phenotype in vascular smooth muscle cells. Circ Res. 1994;74(2):189 196. (10.1161/01.RES.74.2.189) 8293558

[22] Korkmaz M Yakut T Guvenc BH , et al. Comparison of the growth kinetics and morphological characteristics of adult and fetal esophageal smooth muscle cells. Uludag Uni Tip Fak der. 2004;30:27 30.

[23] Morgan JR Yarmush ML , eds. Tissue Engineering Methods and Protocols. Totowa, NJ Humana Press; 1999.

[24] Lazzaro VA Walker RJ Duggin GG Phippard A Horvath JS Tiller DJ . Inhibition of fibroblast proliferation in L-valine reduced selective media. Res Commun Chem Pathol Pharmacol. 1992;75(1):39 48. 1352645

[25] Baskin LS Howard PS Duckett JW Snyder HM Macarak EJ . Bladder smooth muscle cells in culture: I. Identification and characterization. J Urol. 1993;149(1):190 197. (10.1016/s0022-5347(17)36037-8) 8417209

[26] Sharma AK Donovan JL Hagerty JA , et al. Do current bladder smooth muscle cell isolation procedures result in a homogeneous cell population? Implications for bladder tissue engineering. World J Urol. 2009;27(5):687 694. (10.1007/s00345-009-0391-3) 19234706

[27] Bollini S Gentili C Tasso R Cancedda R . The regenerative role of the fetal and adult stem cell secretome. J Clin Med. 2013;2(4):302 327. (10.3390/jcm2040302) 26237150 PMC4470151

[28] Ramkisoensing AA Pijnappels DA Askar SF , et al. Human embryonic and fetal mesenchymal stem cells differentiate toward three different cardiac lineages in contrast to their adult counterparts. PLoS One. 2011;6(9):e24164. (10.1371/journal.pone.0024164) 21931658 PMC3170333

[29] Bi M Yang K Yu T Wu G Li Q . Cell-based mechanisms and strategies of co-culture system both in vivo and vitro for bone tissue engineering. Biomed Pharmacother. 2023;169:115907. (10.1016/j.biopha.2023.115907) 37984308

[30] Dozmorov MG Kropp BP Hurst RE Cheng EY Lin HK . Differentially expressed gene networks in cultured smooth muscle cells from normal and neuropathic bladder. J Smooth Muscle Res. 2007;43(2):55 72. (10.1540/jsmr.43.55) 17598958

[31] Lai JY Yoon CY Yoo JJ Wulf T Atala A . Phenotypic and functionally characterizations of in vivo tissue engineered smooth muscle from normal and pathological bladders. J Urol. 2002;168:1853 7; discussion 1858. (10.1016/S0022-5347(05)64587-7) 12352375

[32] Bowers CW Dahm LM . Maintenance of contractility in dissociated smooth muscle: low-density cultures in a defined medium. Am J Physiol. 1993;264(1 Pt 1):C229 C236. (10.1152/ajpcell.1993.264.1.C229) 8430771

[33] Owens GK . Regulation of differentiation of vascular smooth muscle cells. Physiol Rev. 1995;75(3):487 517. (10.1152/physrev.1995.75.3.487) 7624392

[34] O’Reilly BA Kosaka AH Chang TK , et al. A quantitative analysis of purinoceptor expression in human fetal and adult bladders. J Urol. 2001;165(5):1730 1734. (10.1016/S0022-5347(05)66403-8) 11342965

